# The Lone Lump: Cutaneous Rosai-Dorfman Disease as an Isolated Upper Arm Lesion

**DOI:** 10.7759/cureus.63542

**Published:** 2024-06-30

**Authors:** Ruaa W Al-Hashemi, Shumoos S Aldarraji, Tasnim Abdalla, Sham Hasnah, Ala Abu-Dayeh, Hussam Khalaf Telfah

**Affiliations:** 1 Family Medicine, Primary Health Care Corporation, Doha, QAT; 2 Family Medicine Residency Program, Medical Education, Hamad Medical Corporation, Doha, QAT; 3 Pathology and Laboratory Medicine, Hamad Medical Corporation, Doha, QAT; 4 Histopathology, Hamad General Hospital, Doha, QAT

**Keywords:** treatment, histiocytes, subcutaneous mass, cutaneous, rosai-dorfman disease (rdd)

## Abstract

Rosai-Dorfman disease (RDD) is a rare disorder characterized by excessive growth of histiocytes. We present a case of a 14-year-old female with cutaneous RDD who had a subcutaneous lump on her left arm for three years. The lump became tender and progressively larger over the past year. She had no systemic symptoms, and her physical examination revealed a mobile, tender lump. Laboratory tests were normal. Surgical excision of the lump was performed, and histopathological examination confirmed RDD with the presence of epithelioid histiocytes with eosinophilic and clear cytoplasm, along with emperipolesis and positive staining for CD68, CD163, S100, and OCT2. The patient was referred for follow-up and required no further treatment. RDD can present with subcutaneous masses without systemic symptoms, and it is important to consider RDD in the differential diagnosis of such cases. Surgical excision is the main treatment, and long-term monitoring is necessary due to the potential for disease recurrence. Awareness of cutaneous RDD presentations is crucial for accurate diagnosis and management.

## Introduction

Rosai-Dorfman disease (RDD) is a rare disease that involves increased production and proliferation of histiocytes. It was first recognized in 1965 and has been considered a self-limited disease with an unknown cause, although few patients might have poor outcomes [1,2].

RDD is divided into three types: nodal, extranodal, or mixed. It commonly presents with bilateral cervical lymphadenopathy, mild fever, leukocytosis, and hyperglobulinemia, which can help narrow the physician’s differential diagnosis. The real challenge lies when the disease occurs in other parts of the body, resulting in an extranodal form of the disease, which occurs in 43% of patients [2,3].

Cutaneous RDD accounts for 3% of all RDD cases. It can present with superficial skin lesions or subcutaneous masses without lymph node involvement, which can be misdiagnosed or managed inappropriately as it resembles many other dermatological or cutaneous medical conditions [3,4]. This disorder can sometimes be associated with an autoimmune, hereditary, or malignant pathology; therefore, it is important for physicians to take into consideration cutaneous presentations of this form of the disease to diagnose, manage, and refer appropriately [5].

Here, we report a young female with a cutaneous RDD who presented with a subcutaneous tender lump on the left arm, which may assist other physicians in diagnosing and treating this rare disease.

## Case presentation

A 14-year-old female with no previous medical conditions presented with the chief complaint of a mass on her left upper arm that had been present for the past three years. Initially, the mass was not painful, itchy, or growing. However, approximately one year ago, it became mildly tender and started to enlarge progressively.

The patient denied experiencing any systemic symptoms such as fever, night sweats, fatigue, or myalgia. There was no neck swelling or skin rashes. Additionally, she had no history of headaches, diplopia, or abnormal movements. Furthermore, there was no abdominal pain, nausea, vomiting, or weight loss. The patient had no reported family or personal history of hematologic, autoimmune, or genetic diseases. She did not have a history of taking any oral medications before the appearance of the lesion. There was no family history of a similar presentation and no history of allergy.

**Figure 1:**
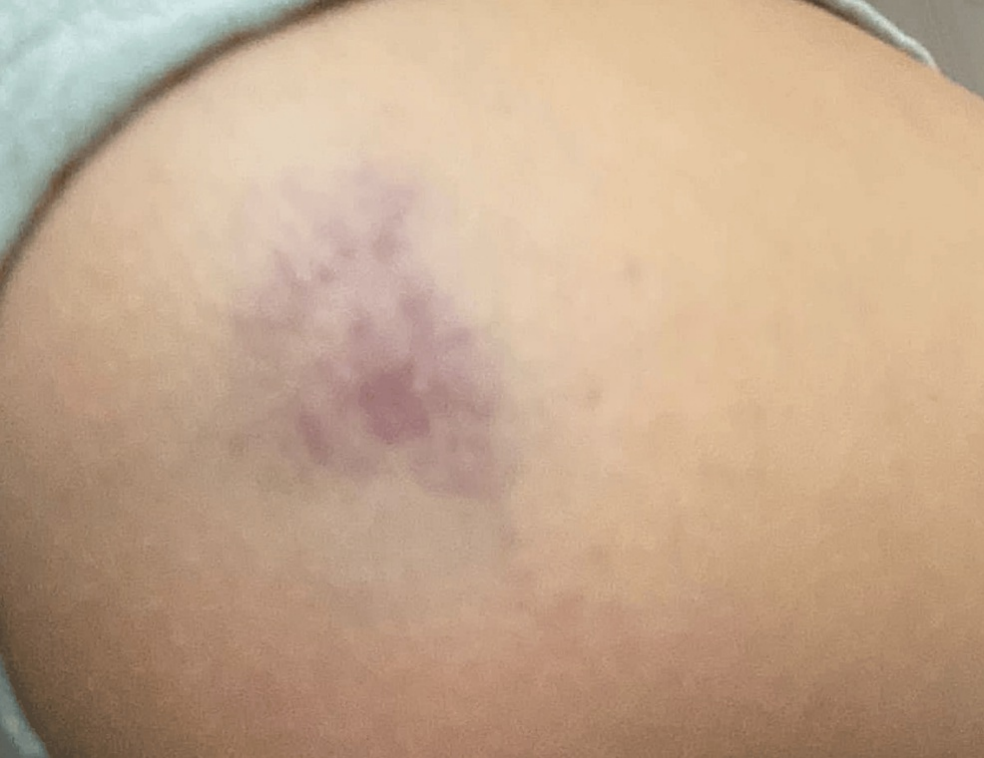
A subcutaneous lump measuring 2 x 3 cm on the left arm is purple in color with a smooth surface

The physical examination revealed no abnormalities in the lung, heart, or abdomen. There were no signs of cervical lymphadenopathy throughout the body and no indications of tonsillar hypertrophy, joint swelling, or tenderness. The skin examination showed a mobile, mildly tender, firm subcutaneous lump measuring 2 x 3 cm on the left arm. The lump had a smooth surface, normal skin temperature, and clear boundaries (Figure 1).

Laboratory investigations, including complete blood counts, renal and liver function tests, electrolytes, ESR, uric acid, LDH, and fibrinogen, all yielded normal results. The peripheral blood smear did not show any abnormalities, and IgA, IgM, and IgG levels were within the normal range.

**Figure 2:**
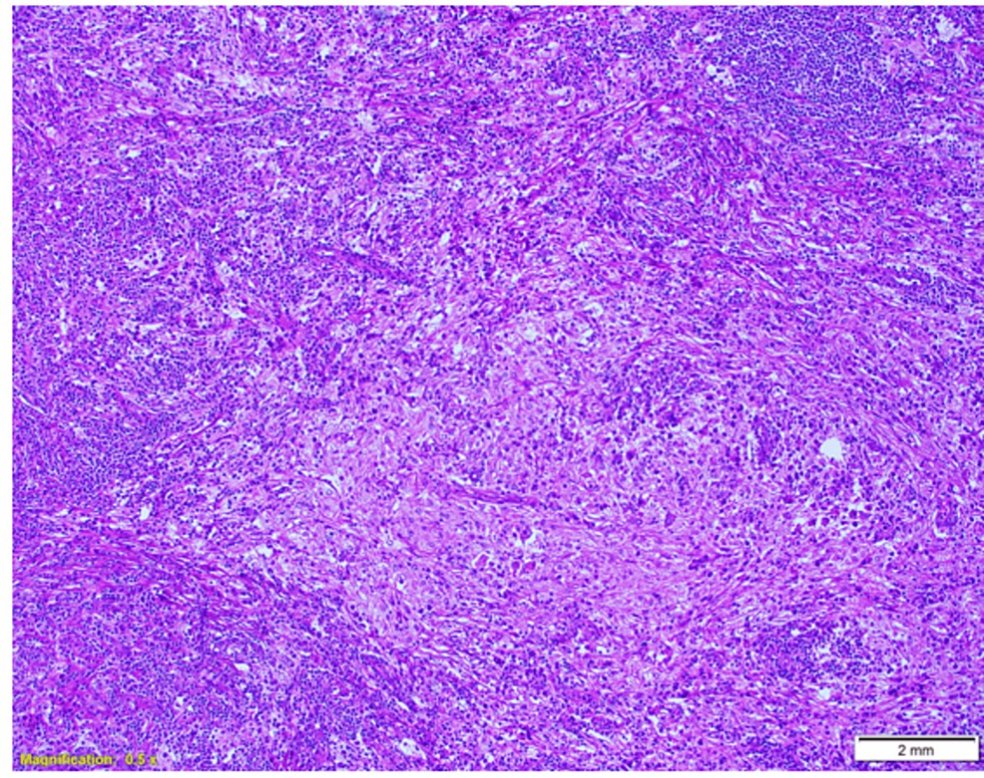
Microscopic and immunohistochemical features of RDD. A photomicrograph shows the proliferation of epithelioid histiocytes with abundant eosinophilic cytoplasm, along with fibrosis and prominent inflammatory infiltrates comprised of plasma cells and lymphocytes

**Figure 3:**
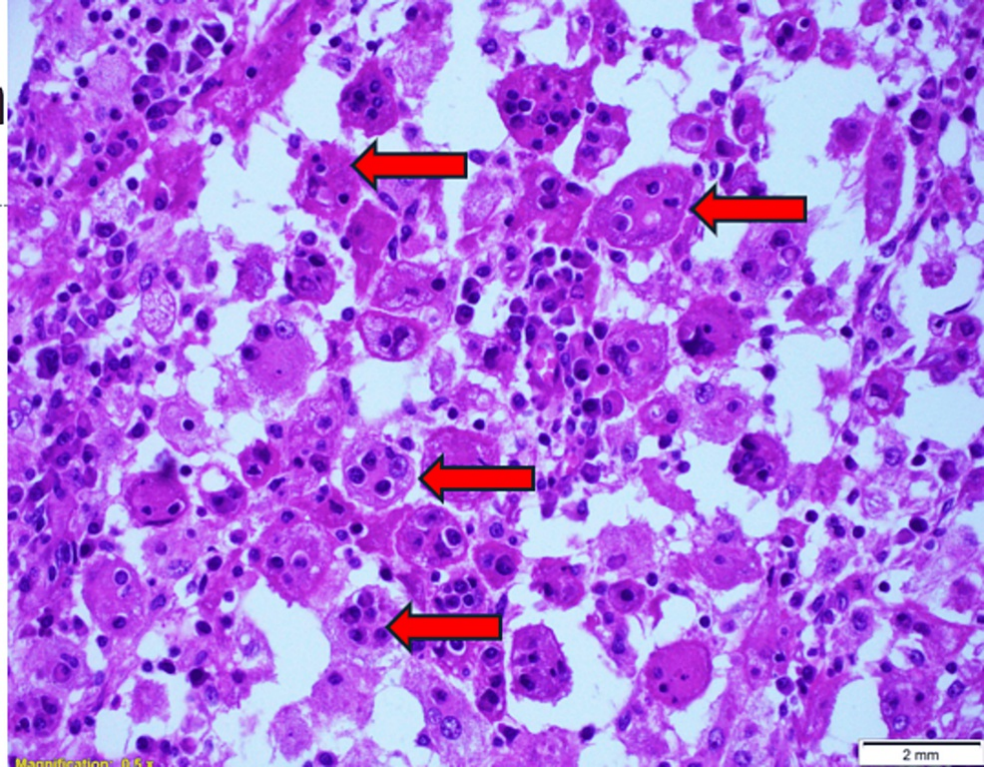
A high-power view demonstrates emperipolesis with the engulfment of inflammatory cells (red arrow)

**Figure 4:**
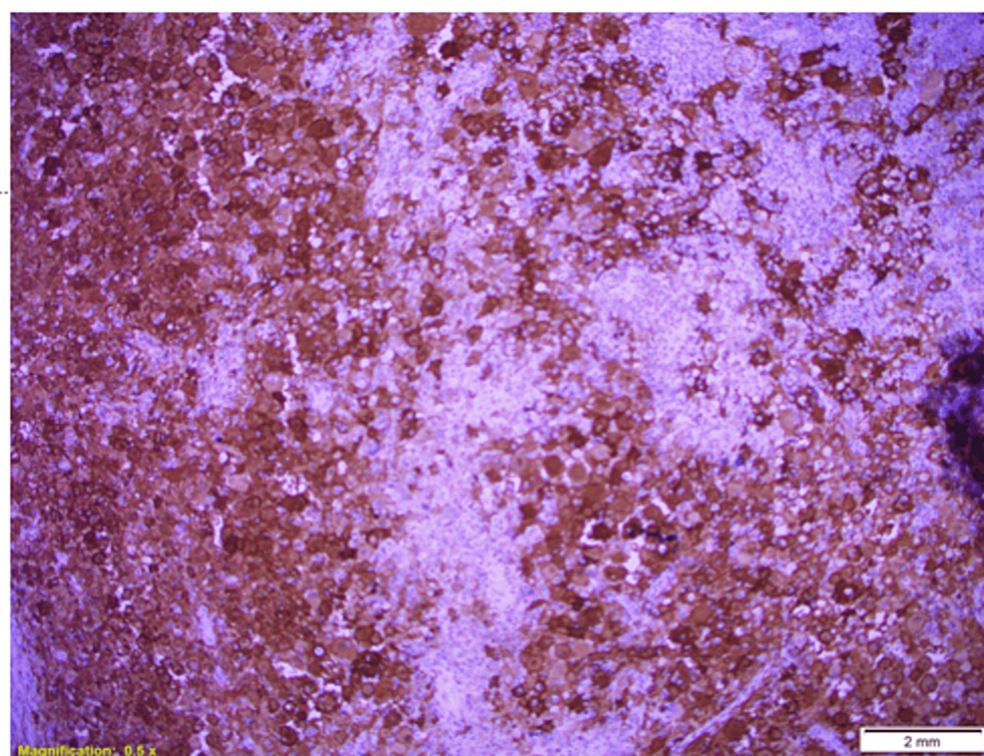
Immunohistochemistry shows that the histocytes are positive for S100

The mass was successfully excised and measured 3.5 x 2.5 x 1 cm in size, consisting of adipose tissue. Microscopic examination revealed a subcutaneous lesion composed of epithelioid histiocytes with eosinophilic and clear cytoplasm (Figure 2). Multinucleated forms and emperipolesis were observed (Figure 3). Immunohistochemistry results showed that the histiocytes were positive for CD68, S100, and OCT2 while negative for Langerin, CD1a, and CK AE1/AE3 (Figure 4). CD3 highlighted T lymphocytes, and CD20 highlighted B lymphocytes. The plasma cells exhibited a polytypic pattern (positive for CD138, Kappa, and Lambda ratios of 2:1). The histopathologist concluded that the findings provided strong evidence supporting a diagnosis of RDD.

The patient was referred to the pediatric hospital's hematology and oncology clinic for additional evaluation and continued care. A whole-body MRI was performed, and it yielded unremarkable results with no notable abnormalities detected, except for an area measuring 2.6 cm in the subcutaneous soft tissues of the medial aspect of the left arm. This area exhibited increased signal intensity, consistent with the previously identified lesion. The neck ultrasound showed the presence of multiple small thyroid colloid cysts, but no lymphadenopathy was observed on either side of the neck. In the clinic, no further treatment was deemed necessary, and the patient was advised to undergo regular observation and follow-up appointments.

## Discussion

RDD is a nonmalignant, rare subtype of non-Langerhans cell histiocytosis that can have a variety of presentations. RDD is mostly seen in children and younger adults, with males more than females. It is also more common in patients of African descent [6]. RDD most commonly presents with painless cervical lymphadenopathy and fever. The most common type of RDD is the classic/nodal RDD, which affects the lymph nodes of the neck [2,3]. RDD can also be present in extranodal sites and can involve different organs in the body. The skin is found to be the most common site of this type of presentation, and the gastrointestinal tract is a rare one [3,7]. In this case, the patient underwent a biopsy to remove a subcutaneous mass. Notably, there was no lymph node involvement or a prior history of fever. This highlights that RDD can manifest as a singular skin lesion without the presence of additional symptoms. Patients with RDD may occasionally exhibit elevated inflammatory markers. However, in the case of our patient, her laboratory results fell within the normal range [3,5,6].

The main diagnostic methods used to identify RDD are immunochemistry and histopathology. RDD is distinguished by the presence of histiocytic infiltration with emperipolesis, which was also observed in our patient's slide. Histiocytes typically exhibit positive staining for markers such as S100 and CD68 while showing negative staining for CD1a. In our patient, the histiocytes displayed positive staining for CD68, CD163, S100, and OCT2 while being negative for CD1a. These findings strongly indicate the presence of RDD [3,8,9]. Various treatment options exist for symptomatic cutaneous RDD, including surgical excision, corticosteroids, thalidomide, cryosurgery, radiotherapy, dapsone, isotretinoin, interferon α, acitretin, and pulsed dye laser. However, their success rates can vary. Surgical excision is commonly considered the first-line treatment for symptomatic RDD, while steroids, chemotherapy, and radiation therapy may be used as additional therapies [10,11]. Although RDD is generally considered a benign condition, there are cases that exhibit aggressive characteristics necessitating systemic medications [12].

A study examining the medical records of 64 RDD patients referred to a tertiary center between 1994 and 2017 discovered that 14% of patients initially presented with asymptomatic RDD but eventually required treatment during follow-up. Among the treatment modalities, surgical excision was the most frequently employed, accounting for 38% of cases. However, within this group, 33% experienced disease relapse.

After the surgical removal of a subcutaneous mass on her arm, our patient was referred to a hematologist/oncologist clinic, which recommended a six-month follow-up period to closely monitor her condition. Additionally, the patient was advised to seek medical attention sooner if the mass reappears or if any other symptoms related to RDD arise.

## Conclusions

We have shed light on the unique presentation of RDD as a subcutaneous mass in a young female without accompanying systemic symptoms. The histopathological and immunohistochemical findings provide compelling evidence supporting the diagnosis, while surgical excision serves as the primary treatment option, especially for symptomatic patients. Long-term monitoring is crucial due to the potential for disease recurrence.
